# Social competence, leisure time activities, and smoking trajectories among adolescent boys: data from the Korean Children & Youth Panel Survey

**DOI:** 10.4178/epih.e2021066

**Published:** 2021-09-09

**Authors:** Eunjung Park, Min Kyung Lim, Jinju Park, Tran Thi Phuong Thao, Sukyung Jeong, Eun Young Park, Jin-Kyoung Oh

**Affiliations:** 1 Division of Cancer Prevention and Early Detection, National Cancer Control Institute, National Cancer Center, Goyang, Korea; 2 Department of Social and Preventive Medicine, Inha University College of Medicine, Incheon, Korea; 3 Central Division of Cardio-Cerebrovascular Disease Management, Seoul National University Hospital, Seoul, Korea; 4 Department of Cancer Control and Population Health, Graduate School of Cancer Science and Policy, National Cancer Center, Goyang, Korea

**Keywords:** Adolescents, Smoking, Trajectory, Reading, Delinquent behaviors, Korea

## Abstract

**OBJECTIVES:**

The purpose of this study was to identify the trajectories and potential predictors of tobacco use during adolescence in Korea and to develop appropriate strategies for the implementation of tobacco use prevention programs.

**METHODS:**

The trajectory of tobacco use and associated predictors were analyzed for 1,169 male students from grade 6 (age 11-12) to grade 10 (age 15-16) in the Korean Children & Youth Panel Survey from 2012 to 2016.

**RESULTS:**

Three trajectories of smoking experience were identified: non-smokers (class 1: n=775, 82.3%), temporary users (class 2: n=32, 3.4%), and regular users (class 3: n=135, 14.3%). When compared to non-smokers, temporary users had a higher likelihood of living with a single parent, dissatisfaction with grades, having a girlfriend, having been victimized at least once, and having at least 1 delinquent friend in grade 7 (when smoking experimentation was at its peak). Significant factors associated with regular use included having a girlfriend, committing at least 1 type of delinquent behavior, and being a non-reader. Committing at least 1 type of delinquent behavior and having at least 1 delinquent friend were associated with regular users, distinguishing them from temporary users.

**CONCLUSIONS:**

Understanding why adolescents exhibit different trajectories of tobacco use by identifying the factors associated with each trajectory can contribute to the development of tailored prevention strategies and early cessation programs for adolescents.

## INTRODUCTION

Most tobacco use begins in childhood or adolescence when an individual is most vulnerable to social influences that promote tobacco use, such as having friends who smoke, engaging in problem behaviors, having a person at home who smokes, policies on sales to minors, smoke-free policies, and tobacco company marketing [1]. Furthermore, initiation of tobacco use at an early age extends the potential duration of tobacco use, leading to stronger nicotine addiction from increased tobacco consumption [2]. Therefore, understanding the course of tobacco use in childhood and adolescence is an integral component of tobacco control and the prevention of lifelong adverse effects on health.

Longitudinal analysis of trajectory data allowed for identification of several different trajectories or subgroups, which were then classified by individual response patterns and finer modeling of individual variations. Analysis results were applied over time to understand factors associated with the different subgroups and to develop prevention strategies that were more tailored to each subgroup [3].

Trajectory analysis from previous studies divided the course of childhood and adolescent tobacco use into several categories such as never-smokers, triers, experimenters, and regular smokers. These studies established common predictors of becoming a sustained smoker through several distinct stages [4-10]. These studies have indicated that childhood and adolescent tobacco use patterns follow different trajectories, involving a complex transition from experimentation to addiction. In addition, variability and inter-individual changes over time in tobacco use behaviors among children and adolescents could be examined. Lastly, factors such as gender, ethnicity, socioeconomic status, academic performance, parent and/or peer tobacco use, tobacco bans at home and/or in public spaces, and perceptions of the tobacco industry have been identified, among others, as predictors of tobacco use trajectories [6,8,9].

However, studies investigating the tobacco use trajectories of children and adolescents have provided insufficient information on (1) the risk factors associated with tobacco use initiation and/or continuation among people of different ethnicities or cultures, (2) the affordability of tobacco use, (3) the prevalence of tobacco use, and (4) tobacco control measures. Furthermore, although lifelong adverse health effects can be expected with a high prevalence of smoking among adolescents, a limited number of studies have explored predictable connections between adolescent health and juvenile delinquency in Asian countries.

The prevalence of smoking among Korean adolescents in 2006 and 2017 was 16.0% and 9.5%, respectively, for boys and 9.2% and 3.1%, respectively, for girls, even though the age for initiating daily cigarette smoking had decreased since 2006 (14.4 and 13.8 years for boys and 14.2 and 13.7 years for girls in 2006 and 2017, respectively) [11]. The decrease in age for initiating daily cigarette consumption emphasizes a need for action to reduce youth access to tobacco products. Therefore, it is important to develop more intensive and effective cessation interventions tailored to the specific needs of smoker subgroups.

Accordingly, the aim of this study was to identify the smoking trajectories and potential predictors of smoking during adolescence in Korea in order to develop appropriate strategies for the primary prevention of tobacco use.

## MATERIALS AND METHODS

### Data and study population

The Korean Children & Youth Panel Survey (KCYPS) began in 2010 with informed consent from each participant. Public data from the KCYPS included a nationally representative, school-based sample of Korean students with stratified multi-stage cluster sampling. The KCYPS, aiming to investigate various aspects of the growth and development of children and youth, started to collect data from 3 different panels, including grade 1 and grade 4 elementary school students and first-year middle school students from 2010 to 2016 (n=7,071). The schools were selected using a probability proportional to size sampling method. The analysis for this study used the grade 4 elementary school student panel (n=2,378) that included participant developmental stages from grade 4 up to 1 year after high school graduation. In wave 7 of data collection, the original sample retention rate of the grade 4 elementary school student panel was 83.2% (n=2,378 in wave 7).

**Figure 1.**
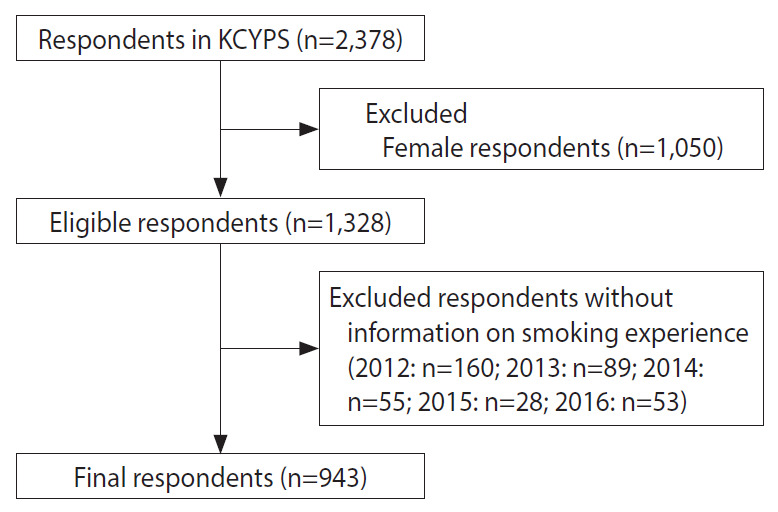
The selection process of respondents included in the final analysis. KCYS, Korean Children & Youth Panel Survey.

The current study included a 5-year (2012 to 2016) follow-up of 1,169 male students from grade 6 (age 11-12) to grade 10 (age 15-16) in the grade 4 panel (age 9-10), because the questionnaire began including questions on smoking experience in grade 6. Of the total 1,169 respondents, 943 were included in the final analysis, after excluding 226 respondents who did not self-report having at least 1 smoking experience and two who had no sampling weight (Figure 1) (Supplementary Material 1).

### Description of study variables

#### Dependent variables

Data for smoking experience, a dichotomous variable (i.e., self-reported experience of smoking during the last year [never/ever]) were collected at each wave.

#### Independent variables

The following factors were examined as predictors of smoking trajectory patterns: family composition (living with both parents and/or grandparents, living with a single parent and/or grandparent), annual family income (< 30,000,000, 30,000,000-55,000,000, ≥ 55,000,000 Korean won), satisfaction with academic grades (satisfied/dissatisfied), having a girlfriend (no/yes), number of days per week without a guardian after school (almost never, 1-2, 3, and > 3 days), having committed at least 1 type of delinquent behavior (never/ever), having been victimized at least once (never/ever), having delinquent friends (none, 1, or more), sleeping time per day (< 8, 8-9, ≥ 9 hours), and leisure activities (reading time per day [never, < 1, ≥ 1 hour] and time spent playing PC/video games per day [< 0.5, 0.5-2, ≥ 2 hours]).

Smoking behavior was measured using respondent self-reports regarding the past year. Satisfaction with the most recent semester grades were ascertained from answers to the questions about family composition at the time of the survey, annual household income for the past year, and the current number of days per week without a guardian after school. Having a girlfriend and participation in delinquent behavior were measured using respondent self-reports for the past year. Delinquent behaviors included violence, smoking, alcohol drinking, unauthorized school absence, teasing or bullying, threatening, stealing, school violence, gang fighting, sexual relations, sexual violence, and gambling. The amount of sleep during weekdays and weekends was calculated using the bedtime and wake-up time questions, with separate questions for weekdays and for weekends. Questions about reading time and PC/video game-playing time were also asked separately for weekdays and for weekends. Sleeping time, reading time, and PC/video game-playing time per day were each calculated as the average amount of time for weekdays and the average amount of time for weekends.

### Statistical analysis

Group-based trajectory models (GBTM) with a group-based and semi-parametric approach were used to identify subgroups that share similar behavioral trajectories for smoking [12,13]. Respondents who reported never having smoked were defined as an a priori group. We fitted the GBTM to male respondents with 1 or more smoking experiences and considered 9 applicant models, allowing 2-4 underlying latent classes with up to 1 cubic polynomial function for each class. The best-fitting model was selected based on the Bayesian information criterion (BIC). These models were fitted using the software SAS Proc Traj [14].

After the number of trajectories was determined, and respondents were assigned to trajectories, simple logistic regression analyses and multiple logistic regression analyses were applied to identify the predictors associated with smoking trajectory patterns. All analyses were conducted separately by year using annually measured predictors. The multiple logistic regression model was applied to the predictors statistically associated with smoking trajectory patterns in the simple logistic regression model. All predictors were mutually adjusted for each other, as there was no multicollinearity when it was calculated based on the variance inflation factor. Logistic regression analyses were performed using the SURVEYLOGISTIC procedure considering a complex sampling design. This panel survey provided 2 kinds of weights: cross-sectional weights and longitudinal weights. Because all analyses, except the trajectory analysis, were carried out separately for each survey year (wave), we selected the cross-sectional weight. All analyses were conducted using SAS version 9.4 (SAS Institute Inc., Cary, NC, USA), and 2-sided p-values < 0.05 were considered to indicate statistical significance.

### Ethics statement

Public data from the 2010 to 2016 waves were used for the cur rent study with institutional review board review approval.

## RESULTS

**Figure 2.**
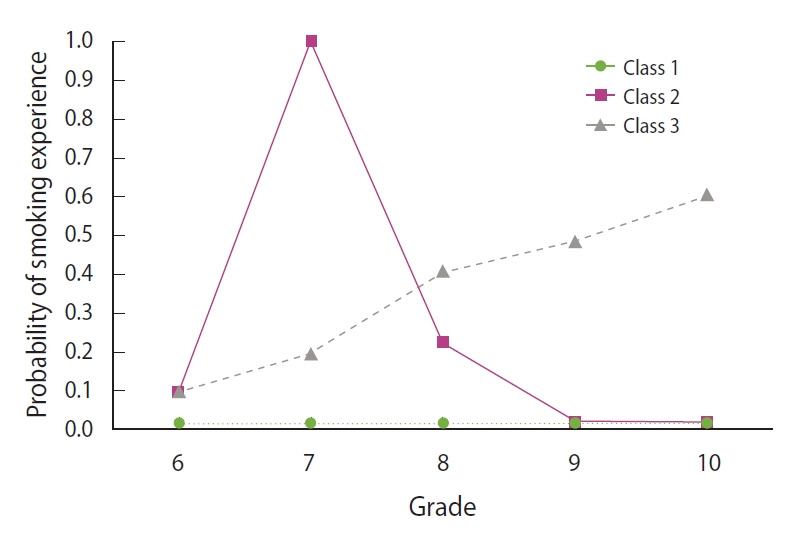
Probability of smoking experience among class 1, 2, and 3 in male adolescent. Class 1, non-users; Class 2, temporary users; Class 3, continuous users.

Respondents who reported never having smoked were specified as an a priori group in the GBTM. A cubic polynomial growth mixture model was finalized with 2 or 3 groups based on having BIC values closest to 0. The 2-group model performed the best statistically (BIC2=-507.72; BIC3=-520.92; BIC4=-533.95) and was selected as the final trajectory model. Figure 2 presents the a priori group and the 2 cigarette smoking trajectories.

The 3 trajectories were named based on their shapes: non-users (class 1: n=775, 82.3%), temporary users (class 2: n=32, 3.4%), and regular users (class 3: n=135, 14.3%). Temporary and regular users were also combined into a single trajectory designated as ever-users (classes 2 and 3 17.7%). The smoking experience rate of experimenters rose sharply between grades 6 and 7, peaked, and then decreased gradually until falling to zero at grade 9. For regular users, the rate of tobacco use began mounting in grade 6 and increased until grade 10 (Figure 2).

Respondent characteristics were compared by grade: ever-smoking experience was 1.7% in grade 6 and regularly increased, reaching 9.1% in grade 10; the percentage of respondents dissatisfied with their grades increased with age; and older respondents spent less time sleeping and reading. However, the percentage of respondents with a girlfriend and at least 1 delinquent friend increased with age. Interestingly, grade 7 had the highest percentage of respondents who had engaged in at least 1 type of delinquent behavior, and grade 6 had the highest percentage of respondents who had been victimized at least once (Table 1).

Four observations were noted when controlling for demographic and adolescent behavioral factors. First, when compared to non-smokers, ever users (temporary and regular users) had a significantly higher likelihood of having a girlfriend, committing at least 1 type of delinquent behavior, and never reading at most grade levels. Dissatisfaction with grades and lower annual household income were associated with being ever-users in grade 6, while having at least 1 delinquent friend in grades 8 and beyond was associated with being an ever-user (Table 2). Second, compared to non-smokers, temporary users had a higher likelihood of living with a single parent in grades 6 and beyond. In grade 7, when smoking experimentation was at its peak, dissatisfaction with grades, having a girlfriend, being victimized at least once, and having at least 1 delinquent friend were identified as factors associated with temporary users. Among temporary users, committing at least 1 type of delinquent behavior in grade 8 and having a girlfriend in grade 9 were also relatively higher (Table 3). Third, when compared to non-smokers, regular users had a higher likelihood of having a girlfriend, committing at least 1 type of delinquent behavior, and never reading in all grades, although never reading showed only a marginal significance in grades 8 and 9. In grade 6, reading less than 1 hour per day and belonging to the lowest household income group (< 30,000,000 Korean won) were significant factors associated with regular use. In grade 8, respondents who self-reported that they were regular users were more likely to have at least 1 delinquent friend (Table 4). Fourth, when compared to temporary users, regular users had a higher likelihood of committing at least 1 type of delinquent behavior and having at least 1 delinquent friend in grades 9 and beyond (Table 5).

## DISCUSSION

This study was the first to identify multiple trajectories of smoking behavior as well as the smoking-related factors associated with each trajectory using a representative sample of Korean adolescents. Therefore, the goals of this analysis were to identify the distinct developmental trajectories of cigarette use spanning adolescence and examine the key risk factors associated with these particular smoking trajectories.

As shown in Table 1 for grade 7 (12-13 years), the number of respondents who reported dissatisfaction with their grades, having a girlfriend, having committed at least 1 type of delinquent behavior, having at least 1 delinquent friend, and making time for reading increased. For this same age group, respondents who were victimized at least once and sleeping time decreased significantly. The number of ever-users also dramatically increased from ages 11-12 (1.7%) to 15-16 (9.1%). These years constitute a critical time during which young people are especially vulnerable due to significant life changes such as starting middle school, increasing academic workloads, and various societal influences (e.g., peer relationships and growing social awareness), all of which can affect their interest in trying, experimenting, and continuing smoking. These results corresponded with the findings of previous studies which investigated the association between the characteristics of puberty and smoking experimentation [2,15]. Therefore, understanding the specific characteristics of individual behaviors and changes in social environments during puberty should be considered when developing an appropriate program to prevent children and adolescents from smoking.

Considering previous studies [1,5,10,15,16], the longitudinal patterns of smoking that were observed in this analysis were best described by 3 smoking trajectory groups: non-smokers (class 1), temporary users (class 2), and regular users (class 3). Compared to non-smokers, the other 2 trajectories were characterized by smoking continuity. Respondents assigned to class 2 (3.4%) started smoking when they turned 12 (the first year of junior high school), their tobacco use peaked at age 13, and then they quit gradually. Individuals assigned to class 3 (14.3%) continued smoking. Distinguishing class 2 from class 3 was of considerable importance for adolescent smoking prevention because it was key to understanding why some individuals discontinue tobacco use while others continue.

Analysis enabled us to examine how smoking risk factors were organized in meaningful patterns that distinguished subgroups of smokers. By incorporating risk factors into the trajectory model, hypotheses that related these risk factors with the probability of inclusion in a trajectory group were tested. Living with a single parent was the significant factor associated with the temporary-user group for all ages. Peak increases in smoking experimentation at age 13 were associated with grade dissatisfaction, having a girlfriend, being victimized at least once, and having at least 1 delinquent friend. At grade 6, belonging to the lowest family income demographic and spending less time reading (never or < 1 hr/d) were identified as significant factors for smoking initiation in the regular-user group, even if the factors did not continue in later ages. Having a girlfriend and having committed at least 1 type of delinquent behavior were significant factors associated with regular use in all grades. These findings suggested that respondents who neither commit delinquent behaviors nor regularly have a girlfriend have a greater possibility of smoking discontinuation, even if smoking experimentation was initiated because of a lack of daily care or parental concern or the societal influence of their peers (e.g., having delinquent friends and being victimized by peers). Furthermore, concern about grades and engaging in positive leisure activities such as reading could be considered factors that help prevent an interest in smoking.

These results were supported by the findings of previous studies published in other countries, including recent studies in the United States and Europe which revealed distinct smoking trajectory patterns and smoking-related factors during adolescence [6,9,14,16-19]. As in this current study, these previous studies suggested that a single-parent family, dating, less sleeping time, delinquent behavior, and poor academic performance were factors associated with smoking initiation and continuation for the period spanning early adolescence to young adulthood. Some of the factors showed a significant association with an increase in adolescent smoking or distinguished smoking trajectories from non-user trajectories. In addition, the current study included new factors such as experiences of victimization, having delinquent friends, absence of a guardian after school, reading time, and PC/video game-playing time. Although previous studies did not include these factors, it was nevertheless important to investigate them in terms of their potential effect on interest in smoking at an early age in adolescence or childhood. The current study also revealed that victimization and reading time were significant factors for distinguishing both temporary users (class 2) and regular users (class 3) from non-smokers (class 1), respectively. The findings are unique and contribute meaningful insights into early adolescent smoking behaviors and explain why different trajectories occur, especially in Asian countries where such information is not available. Furthermore, taking into account that the age of puberty has been decreasing worldwide, including in Europe, early onset of puberty could also be considered a factor associated with early smoking initiation, based on the early maturation hypothesis [20]. Accordingly, the factors included in the current study and their effect on grouping individuals into different smoking trajectories contributed to understanding the influences of the gap between physical maturity and social maturity on health and social behaviors, as well as how these influences relate to smoking initiation and continuation in early adolescence [20,21].

This study differed from previous cross-sectional studies that investigated the factors associated with smoking status and/or behaviors observed in adolescent subjects at a specific time. The current study investigated these factors by analyzing trajectory groups with longitudinal follow-up. Even though results from cross-sectional studies, using data from the Korea Youth Risk Behavior Web-based Survey, suggested that student performance in school, alcohol consumption, and the influence of peer smoking were the factors associated with adolescent tobacco use, the results did not identify the factors that affect which smoking trajectory is followed or which step of the natural history of tobacco use is followed, including never-use, trying, experimentation, and continuation or discontinuation [22]. Whereas, in the current trajectory analysis, factors associated with initiation, continuation, and discontinuation of tobacco use could be identified and fluctuations in their influence tracked over the span of adolescence. For example, living with single parents was a significant factor associated with temporary users through all grades but dissatisfaction with grades, having a girlfriend, being victimized at least once, and having at least 1 delinquent friend were identified as factors associated with temporary users in grade 7 when smoking experimentation was at its peak. Having a girlfriend, committing at least 1 type of delinquent behavior, and never reading were identified as the factors associated with regular users in all grades, but were different for temporary users. These subgroup differences provide significant scientific evidence for developing tailored prevention strategies to address tobacco use in adolescence.

The current study of Korean adolescents offered unique data on smoking trajectories and the factors used to include individuals in different trajectory groups using a representative sample and longitudinal follow-up design. The results were meaningful in terms of understanding the multiple smoking trajectories that exist in early adolescence in relation to certain characteristics of puberty. However, there were a few limitations.

First, although adolescent smoking patterns follow different trajectories and some previous studies have suggested heterogeneity of trajectories in smoking onset and progression, the current study identified 3 kinds of smoking trajectories: non-smokers, temporary users, and regular users [5,15]. In the current study, the 3 kinds of smoking trajectories were retained because heterogeneity did not identify a significantly different trajectory in smoking onset and progression; a statistically best fit for differentiation of the smoking pattern was also shown. In addition, comprehensive, statistically significant comparison of the classes was not possible due to the very small number of subjects in class 2. Second, the current study omitted certain variables that have demonstrated diverse trajectory patterns in previous studies, such as personality, depression, family problems (including parental conflict or violence), school attendance, conduct problems, sibling smoking, parental education level, parental smoking, smoking intensity, and other adolescent behaviors [9,15,16,23-25]. The current study analyzed secondary data from KCYPS; therefore, it was not possible to gather additional information on other variables used in previous studies. In particular, smoking intensity and other more specific smoking behaviors that could further differentiate tobacco users were not considered. Non-smoking policies that affect smoking behaviors were also not included in this study. Variables frequently used in previous studies such as family problems and parental education level might be interchangeable with variables in this study, such as absence of a guardian after school and level of household income. Future studies could also compensate for this limitation by including other variables that have not been considered in previous studies, potentially yielding additional implications. Third, the current analysis was limited to boys. Thus, results for girls were not available. However, as mentioned in the Methods section, this could not be avoided due to the very small number of girls who had ever smoked, thus preventing an appropriate statistical analysis.

In conclusion, this study suggested that adolescent tobacco use in Korea follows 3 distinct smoking trajectories: non-smokers, temporary users, and regular users. Temporary users were associated with living with a single parent. The peak increase in smoking experimentation among grade 7 temporary users was influenced by having a girlfriend, being victimized at least once, and having at least 1 delinquent friend. Having a girlfriend and having committed at least 1 type of delinquent behavior were significant factors for regular users while belonging to the lower family income demographic and spending less time reading at age 12 were significant factors for smoking initiation among regular users. Identification of smoking trajectory patterns and factors associated with inclusion in specific trajectory groups can contribute to the development of prevention and early cessation programs for adolescents tailored to the specific characteristics of subgroups.

## Figures and Tables

**Table 1.** Characteristics of study participants by grade

Characteristics		Grade 6 (11-12 yr)	Grade 7 (12-13 yr)	Grade 8 (13-14 yr)	Grade 9 (14-15 yr)	Grade 10 (15-16 yr)
Smoking experience						
	Ever	16 (1.7)	54 (5.6)	64 (6.7)	69 (7.3)	86 (9.1)
	Never	927 (98.3)	889 (94.4)	879 (93.3)	874 (92.7)	857 (90.9)
Level of satisfaction with grades						
	Satisfied	749 (79.8)	537 (56.7)	520 (55.9)	552 (57.9)	416 (43.3)
	Dissatisfied	192 (20.3)	404 (43.3)	421 (44.0)	387 (42.1)	523 (56.7)
Family composition						
	Living with 2 parents and/or grandparents	844 (90.8)	834 (89.9)	835 (90.4)	828 (89.9)	807 (89.6)
	Living with single parent and/or grandparent	80 (9.1)	86 (10.1)	86 (9.6)	85 (10.1)	84 (10.4)
Annual household income (1,000 Korean won)						
	≥55,000	225 (25.4)	237 (26.3)	246 (27.4)	287 (31.5)	310 (34.9)
	30,000-54,999	529 (55.5)	513 (54.8)	506 (54.4)	483 (51.8)	453 (50.0)
	<30,000	158 (19.0)	155 (18.9)	146 (18.3)	130 (16.7)	113 (15.2)
Have a girlfriend						
	No	809 (85.9)	793 (83.4)	785 (83.4)	762 (81.5)	766 (81.6)
	Yes	131 (14.1)	149 (16.6)	156 (16.6)	180 (18.6)	173 (18.4)
Have committed at least 1 type of delinquent behavior						
	Never	837 (90.0)	761 (79.8)	788 (83.7)	815 (86.2)	761 (81.1)
	Ever	104 (10.0)	181 (20.2)	153 (16.3)	127 (13.8)	178 (18.9)
Have been victimized at least once						
	Never	810 (86.4)	884 (94.1)	894 (95.4)	922 (98.1)	919 (98.0)
	Ever	131 (13.6)	58 (5.8)	47 (4.6)	20 (1.9)	20 (2.0)
Have at least 1 delinquent friend						
	None	741 (79.7)	592 (63.0)	570 (59.2)	575 (61.1)	467 (49.9)
	One or more	201 (20.4)	350 (37.0)	369 (40.8)	364 (38.9)	472 (50.1)
Without a guardian after school (d/wk)						
	Almost never	494 (54.5)	496 (54.8)	463 (52.3)	-	-
	1-2	127 (13.9)	145 (15.1)	194 (19.7)	-	-
	≥3	309 (31.6)	282 (30.0)	267 (28.0)	-	-
Sleeping time (hr/d)						
	≥9	367 (39.1)	153 (17.2)	126 (14.4)	101 (11.6)	32 (3.8)
	≥8-<9	431 (47.3)	448 (48.3)	404 (43.7)	336 (36.4)	114 (13.9)
	<8	127 (13.6)	335 (34.5)	408 (41.9)	505 (52.0)	793 (82.3)
Reading time (hr/d)						
	≥1	269 (29.0)	190 (20.8)	178 (18.9)	194 (19.6)	198 (21.5)
	<1	449 (46.9)	389 (40.0)	374 (39.4)	285 (30.1)	336 (34.3)
	Never	212 (24.2)	351 (39.2)	380 (41.8)	463 (50.3)	405 (44.2)
PC/video game-playing time per day						
	<30 min	111 (11.5)	76 (7.5)	81 (8.4)	103 (10.7)	218 (22.8)
	≥30 min-<2 hr	553 (58.8)	551 (57.0)	494 (52.1)	444 (45.6)	488 (49.8)
	≥2 hr	265 (29.7)	303 (35.6)	358 (39.5)	395 (43.7)	233 (27.3)

Values are presented as unweighted frequency (weighted %).

**Table 2.** Factors associated with ever-users (classes 2 and 3) compared to non-smokers (class 1)

Characteristics		Grade 6 (11-12 yr)		Grade 7 (12-13 yr)		Grade 8 (13-14 yr)		Grade 9 (14-15 yr)		Grade 10 (15-16 yr)	
		Simple	Multiple	Simple	Multiple	Simple	Multiple	Simple	Multiple	Simple	Multiple
Levels of satisfaction with grades											
	Satisfied	1.00 (reference)	-	1.00 (reference)	1.00 (reference)	1.00 (reference)	1.00 (reference)	1.00 (reference)	-	1.00 (reference)	-
	Dissatisfied	1.22 (0.75, 1.97)	-	1.82 (1.26, 2.63)	1.56 (1.03, 2.35)	1.54 (1.04, 2.28)	1.48 (0.96, 2.26)	1.31 (0.89, 1.93)	-	1.05 (0.71, 1.54)	-
Family composition											
	Living with 2 parents and/or grandparents	1.00 (reference)	1.00 (reference)	1.00 (reference)	1.00 (reference)	1.00 (reference)	1.00 (reference)	1.00 (reference)	1.00 (reference)	1.00 (reference)	1.00 (reference)
	Living with single parent and/or grandparent	1.83 (1.03, 3.27)	1.36 (0.71, 2.63)	1.88 (1.05, 3.38)	1.43 (0.71, 2.88)	1.89 (1.03, 3.45)	1.39 (0.69, 2.81)	2.30 (1.20, 4.40)	2.08 (0.96, 4.51)	2.29 (1.34, 3.92)	1.47 (0.81, 2.69)
Annual household income (1,000 Korean won)											
	≥55,000	1.00 (reference)	1.00 (reference)	1.00 (reference)	1.00 (reference)	1.00 (reference)	1.00 (reference)	1.00 (reference)	1.00 (reference)	1.00 (reference)	1.00 (reference)
	30,000-54,999	1.72 (1.13, 2.61)	1.56 (1.03, 2.36)	1.57 (1.01, 2.44)	1.41 (0.88, 2.25)	1.38 (0.88, 2.15)	1.40 (0.86, 2.29)	1.56 (1.00, 2.43)	1.55 (0.99, 2.44)	1.65 (1.05, 2.58)	1.66 (1.00, 2.74)
	<30,000	3.00 (1.60, 5.64)	2.43 (1.26, 4.68)	2.72 (1.37, 5.38)	1.71 (0.81, 3.60)	2.18 (1.18, 4.03)	1.87 (0.97, 3.61)	2.43 (1.23, 4.84)	1.44 (0.74, 2.82)	2.48 (1.30, 4.72)	1.91 (0.95, 3.81)
Have a girlfriend											
	No^1^	1.00 (reference)	1.00 (reference)	1.00 (reference)	1.00 (reference)	1.00 (reference)	1.00 (reference)	1.00 (reference)	1.00 (reference)	1.00 (reference)	1.00 (reference)
	Yes	2.36 (1.46, 3.79)	2.16 (1.35, 3.47)	2.92 (1.87, 4.56)	2.18 (1.39, 3.41)	3.43 (2.29, 5.15)	2.82 (1.77, 4.47)	3.24 (2.13, 4.94)	2.62 (1.66, 4.14)	2.41 (1.58, 3.66)	1.76 (1.06, 2.92)
Have committed at least 1 type of delinquent behavior											
	Never	1.00 (reference)	1.00 (reference)	1.00 (reference)	1.00 (reference)	1.00 (reference)	1.00 (reference)	1.00 (reference)	1.00 (reference)	1.00 (reference)	1.00 (reference)
	Ever	2.20 (1.36, 3.55)	1.73 (0.99, 3.04)	2.84 (1.84, 4.38)	1.90 (1.20, 3.01)	3.88 (2.55, 5.93)	2.75 (1.68, 4.52)	6.37 (4.27, 9.50)	4.39 (2.70, 7.13)	5.99 (4.05, 8.86)	4.26 (2.81, 6.48)
Have been victimized at least once											
	Never	1.00 (reference)	-	1.00 (reference)	-	1.00 (reference)	1.00 (reference)	1.00 (reference)	-	1.00 (reference)	-
	Ever	1.51 (0.79, 2.89)	-	1.85 (0.88, 3.89)	-	2.16 (1.09, 4.27)	0.80 (0.35, 1.81)	0.88 (0.30, 2.62)	-	1.07 (0.32, 3.58)	-
Have at least 1 delinquent friend											
	None	1.00 (reference)	1.00 (reference)	1.00 (reference)	1.00 (reference)	1.00 (reference)	1.00 (reference)	1.00 (reference)	1.00 (reference)	1.00 (reference)	1.00 (reference)
	One or more	1.53 (1.00, 2.33)	1.29 (0.73, 2.28)	1.89 (1.31, 2.71)	1.41 (0.91, 2.19)	2.37 (1.66, 3.38)	1.90 (1.26, 2.88)	2.36 (1.68, 3.30)	1.66 (1.09, 2.53)	3.32 (2.26, 4.88)	2.21 (1.43, 3.44)
Without a guardian after school (d/wk)^1^											
	Almost never	1.00 (reference)	-	1.00 (reference)	-	1.00 (reference)	-	-	-	-	-
	1-2	0.76 (0.42, 1.37)	-	1.02 (0.56, 1.85)	-	1.01 (0.59, 1.74)	-	-	-	-	-
	≥3	1.26 (0.86, 1.86)	-	1.24 (0.80, 1.92)	-	1.30 (0.86, 1.97)	-	-	-	-	-
Sleeping time (hr/d)											
	≥9	1.00 (reference) 1.00 (reference)		1.00 (reference)	-	1.00 (reference)	-	1.00 (reference)	-	1.00 (reference)	-
	≥8-<9	1.52 (1.05, 2.21) 1.67 (1.12, 2.48)		1.39 (0.77, 2.50)	-	0.70 (0.41, 1.19)	-	1.22 (0.62, 2.38)	-	1.67 (0.46, 6.07)	-
	<8	1.82 (1.08, 3.07) 1.73 (0.93, 3.22)		1.17 (0.66, 2.10)	-	0.85 (0.51, 1.43)	-	1.50 (0.78, 2.89)	-	1.61 (0.49, 5.30)	-
Reading time (hr/d)											
	≥1	1.00 (reference) 1.00 (reference)		1.00 (reference)	1.00 (reference)	1.00 (reference)	-	1.00 (reference)	1.00 (reference)	1.00 (reference)	1.00 (reference)
	<1	1.97 (1.26, 3.07) 2.26 (1.37, 3.75)		1.32 (0.71, 2.48)	1.32 (0.69, 2.54)	1.15 (0.66, 2.00)	-	2.01 (1.13, 3.58)	1.07 (0.57, 2.03)	1.20 (0.66, 2.20)	1.13 (0.57, 2.23)
	Never	3.25 (1.85, 5.70) 3.21 (1.63, 6.33)		2.76 (1.56, 4.90)	2.35 (1.32, 4.20)	1.79 (0.96, 3.32)	-	3.38 (1.87, 6.14)	1.99 (1.05, 3.80)	2.76 (1.55, 4.92)	2.35 (1.25, 4.40)
PC/video game-playing time per day											
	<30 min	1.00 (reference)	-	1.00 (reference)	-	1.00 (reference)	-	1.00 (reference)	-	1.00 (reference)	-
	≥30 min-<2 hr	0.92 (0.49, 1.73)	-	0.54 (0.29, 0.99)	-	0.91 (0.45, 1.84)	-	0.80 (0.44, 1.47)	-	1.50 (0.91, 2.47)	-
	≥2 hr	1.40 (0.69, 2.84)	-	0.61 (0.33, 1.12)	-	1.22 (0.61, 2.43)	-	1.45 (0.79, 2.66)	-	1.56 (0.91, 2.67)	-

1 The variable indicating absence of a guardian after school was not collected at grades 9 and 10.

**Table 3.** Factors associated with temporary users (class 2) compared to non-smokers (class 1)

Characteristics		Grade 6 (11-12 yr)		Grade 7 (12-13 yr)		Grade 8 (13-14 yr)		Grade 9 (14-15 yr)		Grade 10 (15-16 yr)	
		Simple	Multiple	Simple	Multiple	Simple	Multiple	Simple	Multiple	Simple	Multiple
Levels of satisfaction with grades											
	Satisfied	1.00 (reference)	-	1.00 (reference)	1.00 (reference)	1.00 (reference)	-	1.00 (reference)	-	1.00 (reference)	-
	Dissatisfied	1.15 (0.52, 2.54)	-	2.63 (1.21, 5.69)	3.03 (1.34, 6.89)	1.79 (0.75, 4.26)	-	1.54 (0.68, 3.48)	-	0.97 (0.43, 2.18)	-
Family composition											
	Living with 2 parents and/or grandparents	1.00 (reference)	1.00 (reference)	1.00 (reference)	1.00 (reference)	1.00 (reference)	1.00 (reference)	1.00 (reference)	1.00 (reference)	1.00 (reference)	1.00 (reference)
	Living with single parent and/or grandparent	4.23 (1.35, 13.3)	3.56 (1.23, 10.3)	4.53 (1.70, 12.1)	4.00 (1.38, 11.6)	5.54 (2.01, 15.2)	4.48 (1.58, 12.7)	6.26 (2.36, 16.6)	4.92 (1.97, 12.3)	6.31 (2.57, 15.5)	3.52 (1.58, 7.86)
Annual household income (1,000 Korean won)											
	≥55,000	1.00 (reference)	1.00 (reference)	1.00 (reference)	1.00 (reference)	1.00 (reference)	1.00 (reference)	1.00 (reference)	1.00 (reference)	1.00 (reference)	1.00 (reference)
	30,000-54,999	1.99 (0.79, 4.99)	2.09 (0.83, 5.24)	1.30 (0.44, 3.82)	0.96 (0.28, 3.23)	1.58 (0.65, 3.88)	1.32 (0.48, 3.62)	1.91 (0.79, 4.63)	1.58 (0.63, 3.98)	2.04 (0.70, 5.97)	1.68 (0.58, 4.89)
	<30,000	4.65 (1.23, 17.7)	2.73 (0.81, 9.20)	3.05 (0.84, 11.1)	0.85 (0.22, 3.33)	3.90 (1.21, 12.6)	1.82 (0.55, 6.03)	5.02 (1.54, 16.3)	1.85 (0.52, 6.55)	5.91 (1.72, 20.3)	2.38 (0.76, 7.53)
Have a girlfriend											
	No	1.00 (reference)	1.00 (reference)	1.00 (reference)	1.00 (reference)	1.00 (reference)	1.00 (reference)	1.00 (reference)	1.00 (reference)	1.00 (reference)	1.00 (reference)
	Yes	2.79 (1.11, 6.99)	2.37 (0.86, 6.56)	3.78 (1.52, 9.43)	2.42 (1.02, 5.73)	2.81 (1.23, 6.45)	2.05 (0.75, 5.58)	3.50 (1.51, 8.12)	3.73 (1.53, 9.10)	3.07 (1.33, 7.08)	2.31 (0.90, 5.93)
Have committed at least 1 type of delinquent behavior											
	Never	1.00 (reference)	1.00 (reference)	1.00 (reference)	1.00 (reference)	1.00 (reference)	1.00 (reference)	1.00 (reference)	1.00 (reference)	1.00 (reference)	1.00 (reference)
	Ever	0.31 (0.04, 2.36)	0.26 (0.03, 2.09)	5.97 (2.36, 15.08)	1.98 (0.80, 4.88)	4.09 (1.85, 9.04)	4.13 (1.84, 9.27)	0.73 (0.16, 3.37)	0.19 (0.02, 2.17)	1.23 (0.42, 3.59)	1.05 (0.28, 3.92)
Have been victimized at least once											
	Never	1.00 (reference)	-	1.00 (reference)	1.00 (reference)	1.00 (reference)	1.00 (reference)	-	-	-	-
	Ever	1.18 (0.26, 5.29)	-	4.59 (1.36, 15.5)	5.05 (1.67, 15.2)	1.13 (0.25, 5.10)	0.36 (0.07, 1.74)	NA	NA	NA	NA
Have at least 1 delinquent friend											
	None	1.00 (reference)	1.00 (reference)	1.00 (reference)	1.00 (reference)	1.00 (reference)	1.00 (reference)	1.00 (reference)	1.00 (reference)	1.00 (reference)	1.00 (reference)
	One or more	0.86 (0.30, 2.44)	1.17 (0.36, 3.83)	11.88 (4.90, 28.8)	8.75 (3.79, 20.2)	1.89 (0.88, 4.05)	1.47 (0.63, 3.41)	0.53 (0.14, 2.00)	0.78 (0.20, 3.05)	0.61 (0.26, 1.45)	0.58 (0.21, 1.61)
Without a guardian after school (d/wk)^1^											
	Almost never	1.00 (reference)	-	1.00 (reference)	-	1.00 (reference)	-	-	-	-	-
	1-2	0.80 (0.18, 3.61)	-	0.55 (0.19, 1.66)	-	0.72 (0.18, 2.87)	-	-	-	-	-
	≥3	1.38 (0.64, 2.97)	-	1.02 (0.45, 2.27)	-	0.91 (0.40, 2.05)	-	-	-	-	-
Sleeping time (hr/d)											
	≥9	1.00 (reference)	1.00 (reference)	1.00 (reference)	-	1.00 (reference)	-	1.00 (reference)	-	-	-
	≥8-<9	2.97 (1.09, 8.10)	3.70 (1.30, 10.5)	2.43 (0.52, 11.4)	-	0.42 (0.13, 1.32)	-	2.00 (0.40, 10.0)	-	NA	NA
	<8	2.72 (0.85, 8.72)	2.76 (0.69, 11.0)	2.01 (0.41, 9.92)	-	0.79 (0.24, 2.61)	-	2.24 (0.51, 9.82)	-	NA	NA
Reading time (hr/d)											
	≥1	1.00 (reference)	1.00 (reference)	1.00 (reference)	1.00 (reference)	1.00 (reference)	1.00 (reference)	1.00 (reference)	1.00 (reference)	1.00 (reference)	1.00 (reference)
	<1	1.79 (0.62, 5.15)	1.49 (0.48, 4.67)	1.16 (0.26, 5.15)	0.91 (0.22, 3.70)	0.66 (0.19, 2.30)	0.63 (0.20, 1.99)	3.25 (0.93, 11.4)	0.99 (0.24, 4.11)	0.47 (0.15, 1.51)	0.45 (0.11, 1.80)
	Never	3.67 (1.17, 11.5)	3.07 (0.85, 11.1)	2.99 (0.90, 9.89)	3.28 (0.92, 11.7)	1.22 (0.35, 4.24)	1.05 (0.33, 3.33)	2.24 (0.61, 8.24)	2.19 (0.62, 7.70)	1.50 (0.56, 3.96)	1.37 (0.49, 3.79)
PC/video game-playing time per day											
	<30 min	1.00 (reference)	-	1.00 (reference)	-	1.00 (reference)	-	1.00 (reference)	-	1.00 (reference)	-
	≥30 min-<2 hr	0.49 (0.20, 1.22)	-	0.57 (0.16, 2.06)	-	0.35 (0.08, 1.54)	-	1.34 (0.33, 5.33)	-	1.30 (0.50, 3.35)	-
	≥2 hr	1.23 (0.45, 3.38)	-	0.56 (0.16, 2.02)	-	0.69 (0.20, 2.45)	-	2.58 (0.73, 9.16)	-	1.70 (0.65, 4.44)	-

NA, not available.

1 The variable indicating absence of a guardian after school was not collected at grades 9 and 10.

**Table 4.** Factors associated with regular users (class 3) compared to non-smokers (class 1)

Characteristics		Grade 6 (11-12 yr)		Grade 7 (12-13 yr)		Grade 8 (13-14 yr)		Grade 9 (14-15 yr)		Grade 10 (15-16 yr)	
		Simple	Multiple	Simple	Multiple	Simple	Multiple	Simple	Multiple	Simple	Multiple
Levels of satisfaction with grades											
	Satisfied	1.00 (reference)	-	1.00 (reference)	1.00 (reference)	1.00 (reference)	-	1.00 (reference)	-	1.00 (reference)	-
	Dissatisfied	1.23 (0.73, 2.08)	-	1.68 (1.13, 2.48)	1.42 (0.93, 2.16)	1.48 (0.99, 2.24)	-	1.26 (0.82, 1.92)	-	1.07 (0.70, 1.63)	-
Family composition											
	Living with 2 parents and/or grandparents	1.00 (reference)	1.00 (reference)	1.00 (reference)	1.00 (reference)	1.00 (reference)	1.00 (reference)	1.00 (reference)	1.00 (reference)	1.00 (reference)	1.00 (reference)
	Living with single parent and/or grandparent	1.38 (0.74, 2.56)	0.98 (0.52, 1.85)	1.38 (0.74, 2.57)	1.11 (0.54, 2.27)	1.29 (0.67, 2.50)	1.07 (0.50, 2.27)	1.64 (0.74, 3.61)	1.38 (0.55, 3.42)	1.66 (0.87, 3.16)	1.03 (0.53, 1.98)
Annual household income (1,000 Korean won)											
	≥55,000	1.00 (reference)	1.00 (reference)	1.00 (reference)	1.00 (reference)	1.00 (reference)	1.00 (reference)	1.00 (reference)	1.00 (reference)	1.00 (reference)	1.00 (reference)
	30,000-54,999	1.67 (1.05, 2.66)	1.47 (0.92, 2.34)	1.64 (1.06, 2.54)	1.53 (0.97, 2.41)	1.34 (0.83, 2.17)	1.36 (0.79, 2.34)	1.50 (0.92, 2.44)	1.59 (0.97, 2.62)	1.59 (0.99, 2.56)	1.67 (0.96, 2.91)
	<30,000	2.71 (1.36, 5.43)	2.40 (1.19, 4.83)	2.63 (1.26, 5.49)	1.89 (0.88, 4.06)	1.89 (0.96, 3.72)	1.66 (0.80, 3.42)	2.04 (0.91, 4.57)	1.35 (0.62, 2.90)	1.99 (0.93, 4.26)	1.78 (0.79, 3.99)
Have a girlfriend											
	No	1.00 (reference)	1.00 (reference)	1.00 (reference)	1.00 (reference)	1.00 (reference)	1.00 (reference)	1.00 (reference)	1.00 (reference)	1.00 (reference)	1.00 (reference)
	Yes	2.26 (1.29, 3.96)	2.12 (1.21, 3.71)	2.73 (1.65, 4.52)	2.21 (1.35, 3.60)	3.59 (2.34, 5.52)	3.17 (1.97, 5.10)	3.19 (2.06, 4.93)	2.41 (1.52, 3.83)	2.27 (1.45, 3.53)	1.62 (0.92, 2.84)
Have committed at least 1 type of delinquent behavior											
	Never	1.00 (reference)	1.00 (reference)	1.00 (reference)	1.00 (reference)	1.00 (reference)	1.00 (reference)	1.00 (reference)	1.00 (reference)	1.00 (reference)	1.00 (reference)
	Ever	2.75 (1.64, 4.60)	2.14 (1.14, 4.02)	2.34 (1.47, 3.74)	1.69 (1.03, 2.78)	3.84 (2.39, 6.14)	2.38 (1.40, 4.04)	8.67 (5.71, 13.2)	5.70 (3.63, 8.98)	8.09 (5.30, 12.3)	5.14 (3.32, 7.95)
Have been victimized at least once											
	Never	1.00 (reference)	-	1.00 (reference)	1.00 (reference)	1.00 (reference)	1.00 (reference)	1.00 (reference)	-	1.00 (reference)	-
	Ever	1.59 (0.90, 2.83)	-	1.29 (0.56, 2.94)	1.64 (0.66, 4.09)	2.41 (1.17, 4.97)	0.91 (0.38, 2.15)	1.10 (0.37, 3.26)	-	1.33 (0.40, 4.44)	-
Have at least 1 delinquent friend											
	None	1.00 (reference)	1.00 (reference)	1.00 (reference)	1.00 (reference)	1.00 (reference)	1.00 (reference)	1.00 (reference)	1.00 (reference)	1.00 (reference)	1.00 (reference)
	One or more	1.71 (1.13, 2.59)	1.29 (0.70, 2.38)	1.33 (0.89, 1.99)	0.98 (0.61, 1.58)	2.50 (1.71, 3.67)	2.05 (1.29, 3.25)	3.30 (2.28, 4.79)	2.02 (1.26, 3.23)	5.79 (3.51, 9.54)	3.51 (2.02, 6.10)
Without a guardian after school (d/wk)^1^											
	Almost never	1.00 (reference)	-	1.00 (reference)	-	1.00 (reference)	-	-	-	-	-
	1-2	0.75 (0.38, 1.48)	-	1.15 (0.61, 2.20)	-	1.09 (0.64, 1.86)	-	-	-	-	-
	≥3	1.24 (0.82, 1.87)	-	1.31 (0.81, 2.10)	-	1.41 (0.89, 2.22)	-	-	-	-	-
Sleeping time (hr/d)											
	≥9	1.00 (reference)	1.00 (reference)	1.00 (reference)	-	1.00 (reference)	-	1.00 (reference)	-	1.00 (reference)	-
	≥8-<9	1.31 (0.89, 1.94)	1.42 (0.92, 2.18)	1.26 (0.70, 2.25)	-	0.78 (0.42, 1.43)	-	1.10 (0.54, 2.24)	-	1.24 (0.33, 4.62)	-
	<8	1.69 (0.95, 3.02)	1.63 (0.82, 3.21)	1.06 (0.59, 1.93)	-	0.87 (0.50, 1.51)	-	1.40 (0.70, 2.78)	-	1.31 (0.40, 4.32)	-
Reading time (hr/d)											
	≥1	1.00 (reference)	1.00 (reference)	1.00 (reference)	1.00 (reference)	1.00 (reference)	1.00 (reference)	1.00 (reference)	1.00 (reference)	1.00 (reference)	1.00 (reference)
	<1	2.01 (1.21, 3.35)	2.47 (1.37, 4.44)	1.36 (0.70, 2.64)	1.32 (0.66, 2.63)	1.33 (0.72, 2.44)	1.43 (0.74, 2.75)	1.69 (0.84, 3.40)	1.10 (0.55, 2.17)	1.58 (0.75, 3.29)	1.42 (0.65, 3.12)
	Never	3.15 (1.71, 5.78)	3.24 (1.58, 6.65)	2.71 (1.42, 5.19)	2.30 (1.22, 4.37)	1.99 (1.04, 3.84)	1.88 (0.94, 3.74)	3.68 (1.94, 7.00)	1.97 (0.96, 4.07)	3.41 (1.67, 6.93)	2.79 (1.33, 5.86)
PC/video game-playing time per day											
	<30 min	1.00 (reference)	-	1.00 (reference)	-	1.00 (reference)	-	1.00 (reference)	-	1.00 (reference)	-
	≥30 min-<2 hr	1.08 (0.54, 2.18)	-	0.53 (0.27, 1.04)	-	1.21 (0.56, 2.62)	-	0.73 (0.39, 1.37)	-	1.55 (0.88, 2.72)	-
	≥2 hr	1.46 (0.67, 3.19)	-	0.62 (0.31, 1.24)	-	1.51 (0.71, 3.18)	-	1.30 (0.68, 2.52)	-	1.53 (0.82, 2.85)	-

1 The variable indicating absence of a guardian after school was not collected at grades 9 and 10.

**Table 5.** The factors associated with regular users (class 3) compared to temporary users (class 2)

Characteristics		Grade 6 (11-12 yr)		Grade 7 (12-13 yr)		Grade 8 (13-14 yr)		Grade 9 (14-15 yr)		Grade 10 (15-16 yr)	
		Simple	Multiple	Simple	Multiple	Simple	Multiple	Simple	Multiple	Simple	Multiple
Levels of satisfaction with grades											
	Satisfied	1.00 (reference)	-	1.00 (reference)	1.00 (reference)	1.00 (reference)	-	1.00 (reference)	-	1.00 (reference)	-
	Dissatisfied	1.07 (0.45, 2.53)	-	0.64 (0.28, 1.46)	0.47 (0.20, 1.09)	0.83 (0.34, 2.06)	-	0.82 (0.34, 1.98)	-	1.11 (0.45, 2.71)	-
Family composition											
	Living with 2 parents and/or grandparents	1.00 (reference)	1.00 (reference)	1.00 (reference)	1.00 (reference)	1.00 (reference)	1.00 (reference)	1.00 (reference)	1.00 (reference)	1.00 (reference)	1.00 (reference)
	Living with single parent and/or grandparent	0.33 (0.09, 1.13)	0.28 (0.10, 0.78)	0.31 (0.11, 0.83)	0.28 (0.10, 0.79)	0.23 (0.08, 0.69)	0.24 (0.09, 0.67)	0.26 (0.09, 0.81)	0.27 (0.09, 0.85)	0.26 (0.09, 0.76)	0.29 (0.11, 0.75)
Annual household income (1,000 Korean won)											
	≥55,000	1.00 (reference)	1.00 (reference)	1.00 (reference)	1.00 (reference)	1.00 (reference)	1.00 (reference)	1.00 (reference)	1.00 (reference)	1.00 (reference)	1.00 (reference)
	30,000-54,999	0.84 (0.30, 2.36)	0.70 (0.25, 1.94)	1.26 (0.41, 3.84)	1.60 (0.47, 5.47)	0.85 (0.32, 2.24)	1.03 (0.34, 3.07)	0.78 (0.29, 2.10)	0.93 (0.34, 2.55)	0.78 (0.25, 2.43)	1.00 (0.32, 3.17)
	<30,000	0.58 (0.14, 2.46)	0.88 (0.24, 3.22)	0.86 (0.22, 3.37)	2.23 (0.56, 8.85)	0.49 (0.14, 1.68)	0.91 (0.25, 3.31)	0.41 (0.11, 1.58)	0.60 (0.17, 2.11)	0.34 (0.08, 1.36)	0.75 (0.20, 2.79)
Have a girlfriend											
	No	1.00 (reference)	1.00 (reference)	1.00 (reference)	1.00 (reference)	1.00 (reference)	1.00 (reference)	1.00 (reference)	1.00 (reference)	1.00 (reference)	1.00 (reference)
	Yes	0.81 (0.28, 2.38)	0.89 (0.26, 3.02)	0.72 (0.26, 1.99)	0.91 (0.37, 2.27)	1.28 (0.53, 3.07)	1.55 (0.55, 4.34)	0.91 (0.38, 2.17)	0.65 (0.28, 1.54)	0.74 (0.31, 1.78)	0.70 (0.25, 2.01)
Have committed at least 1 type of delinquent behavior											
	Never	1.00 (reference)	1.00 (reference)	1.00 (reference)	1.00 (reference)	1.00 (reference)	1.00 (reference)	1.00 (reference)	1.00 (reference)	1.00 (reference)	1.00 (reference)
	Ever	8.81 (1.10, 70.78)	8.08 (0.89, 73.59)	0.39 (0.15, 1.01)	0.86 (0.34, 2.17)	0.94 (0.39, 2.28)	0.58 (0.24, 1.37)	11.90 (2.47, 57.29)	26.62 (2.51, 282.90)	6.60 (2.17, 20.08)	4.91 (1.28, 18.78)
Have been victimized at least once											
	Never	1.00 (reference)	-	1.00 (reference)	1.00 (reference)	1.00 (reference)	1.00 (reference)	-	-	-	-
	Ever	1.35 (0.38, 4.84)	-	0.28 (0.07, 1.11)	0.33 (0.10, 1.12)	2.14 (0.43, 10.59)	2.53 (0.50, 12.92)	-	-	-	-
Have at least 1 delinquent friend											
	None	1.00 (reference)	1.00 (reference)	1.00 (reference)	1.00 (reference)	1.00 (reference)	1.00 (reference)	1.00 (reference)	1.00 (reference)	1.00 (reference)	1.00 (reference)
	One or more	2.00 (0.69, 5.78)	1.11 (0.32, 3.87)	0.11 (0.04, 0.29)	0.11 (0.05, 0.28)	1.33 (0.58, 3.03)	1.40 (0.55, 3.55)	6.23 (1.54, 25.14)	2.55 (0.58, 11.23)	9.50 (3.56, 25.36)	6.01 (1.96, 18.41)
Without a guardian after school (d/wk)^1^											
	Almost never	1.00 (reference)	-	1.00 (reference)	-	1.00 (reference)	-	-	-	-	-
	1-2	0.94 (0.17, 5.27)	-	2.08 (0.64, 6.76)	-	1.51 (0.39, 5.78)	-	-	-	-	-
	≥3	0.90 (0.40, 1.99)	-	1.29 (0.54, 3.09)	-	1.55 (0.62, 3.87)	-	-	-	-	-
Sleeping time (hr/d)											
	≥9	1.00 (reference)	1.00 (reference)	1.00 (reference)	-	1.00 (reference)		1.00 (reference)	-	-	-
	≥8-<9	0.44 (0.15, 1.28)	0.38 (0.13, 1.18)	0.52 (0.12, 2.32)	-	1.85 (0.49, 6.98)		0.55 (0.10, 2.98)	-	-	-
	<8	0.62 (0.17, 2.25)	0.59 (0.13, 2.67)	0.53 (0.11, 2.59)	-	1.10 (0.30, 4.00)		0.62 (0.13, 2.91)	-	-	-
Reading time (hr/d)											
	≥1	1.00 (reference)	1.00 (reference)	1.00 (reference)	1.00 (reference)	1.00 (reference)	1.00 (reference)	1.00 (reference)	1.00 (reference)	1.00 (reference)	1.00 (reference)
	<1	1.13 (0.34, 3.75)	1.65 (0.45, 6.09)	1.17 (0.24, 5.72)	1.46 (0.34, 6.32)	2.03 (0.51, 7.99)	2.27 (0.64, 8.05)	0.52 (0.12, 2.27)	0.80 (0.19, 3.39)	3.33 (0.83, 13.38)	3.20 (0.64, 16.01)
	Never	0.86 (0.25, 2.96)	1.04 (0.27, 4.15)	0.91 (0.23, 3.57)	0.70 (0.17, 2.90)	1.63 (0.43, 6.23)	1.79 (0.51, 6.25)	1.65 (0.40, 6.85)	1.94 (0.44, 8.52)	2.28 (0.69, 7.50)	2.04 (0.60, 6.99)
PC/video game-playing time per day											
	<30 min	1.00 (reference)	-	1.00 (reference)	-	1.00 (reference)	-	1.00 (reference)	-	1.00 (reference)	-
	≥30 min-<2 hr	2.20 (0.82, 5.94)	-	0.93 (0.23, 3.80)	-	3.46 (0.64, 18.61)	-	0.55 (0.13, 2.26)	-	1.19 (0.41, 3.50)	-
	≥2 hr	1.18 (0.40, 3.51)	-	1.11 (0.26, 4.65)	-	2.17 (0.52, 9.05)	-	0.51 (0.13, 1.98)	-	0.90 (0.29, 2.80)	-

1 The variable indicating absence of a guardian after school was not collected at grades 9 and 10.

## SUPPLEMENTARY MATERIALS

Supplementary Material 1. Characteristics of study participants and excluded participants at grade 6
