# Supplementary material for: Social competence, leisure time activities, and smoking trajectories among adolescent boys: data from the Korean Children & Youth Panel Survey
Source: Epidemiol Health. 2021 Sep 9;43:e2021066. doi: 10.4178/epih.e2021066 (PMC8850948; doi:10.4178/epih.e2021066)
Supplement: Supplementary Material 1. — Characteristics of study participants and excluded participants at grade 6 [file epih-43-e2021066-suppl.docx]

. Supplementary Material 1. Characteristics of study participants and excluded participants at grade 6

| Characteristics | | Study participants | Excluded participants |  |
| --- | --- | --- | --- | --- |
|  | | N (%) ^1^ | | *P*-value ^2^ |
| Level of satisfaction with grades | |  |  |  |
|  | Satisfied | 749 (79.8) | 176 (77.1) | 0.3002 |
|  | Dissatisfied | 192 (20.3) | 48 (22.9) |  |
| Family composition | |  |  |  |
|  | Living with 2 parents and/or grandparents | 844 (90.8) | 191 (92.6) | 0.6672 |
|  | Living with single parent and/or grandparent | 80 (9.1) | 18 (7.4) |  |
| Annual household income (1,000 KRW) | |  |  |  |
|  | 55,000 and over | 225 (25.4) | 62 (28.8) | 0.3187 |
|  | 30,000 to 54,999 | 529 (55.5) | 97 (49.1) |  |
|  | Under 30,000 | 158 (19.0) | 45 (22.1) |  |
| Have a girlfriend | |  |  |  |
|  | No | 809 (85.9) | 198 (87.5) | 0.3225 |
|  | Yes | 131 (14.1) | 27 (12.5) |  |
| Have committed at least 1 type of delinquent behavior | |  |  |  |
|  | Never | 837 (90.0) | 195 (88.8) | 0.5942 |
|  | Ever | 104 (10.0) | 29 (11.2) |  |
| Have been victimized at least once | |  |  |  |
|  | Never | 810 (86.4) | 192 (84.1) | 0.4358 |
|  | Ever | 131 (13.6) | 33 (15.9) |  |
| Have at least 1 delinquent friend | |  |  |  |
|  | None | 741 (79.7) | 175 (78.4) | 0.6806 |
|  | One and more | 201 (20.4) | 50 (21.6) |  |
| Number of days per week without a  guardian after school | |  |  |  |
|  | Almost never | 494 (54.5) | 127 (61.9) | 0.1541 |
|  | 1-2 days | 127 (13.9) | 26 (11.7) |  |
|  | 3 days and over | 309 (31.6) | 57 (26.4) |  |
| Sleeping time per day | |  |  |  |
|  | ≥ 9 hours | 367 (39.1) | 91 (39.9) | 0.8522 |
|  | ≥ 8 and < 9 hours | 431 (47.3) | 102 (48.0) |  |
|  | < 8 hours | 127 (13.6) | 29 (12.1) |  |
| Reading time per day | |  |  |  |
|  | ≥ 1 hour | 269 (29.0) | 66 (32.1) | 0.7047 |
|  | < 1 hour | 449 (46.9) | 106 (44.7) |  |
|  | Never | 212 (24.2) | 50 (23.2) |  |
| PC/video game-playing time per day | |  |  |  |
|  | < 30 minutes | 111 (11.5) | 35 (15.9) | 0.2501 |
|  | ≥ 30 minutes and < 2 hours | 553 (58.8) | 128 (55.4) |  |
|  | ≥ 2 hours | 265 (29.7) | 59 (28.7) |  |

^1^N means unweighted frequency and % means weighted percent

^2^The Rao-Scotti chi-square test was performed to examine difference between study participants and excluded participants.
